# Genetic Influences of Proinflammatory Cytokines on Pain Severity in Patients with Temporomandibular Disorders

**DOI:** 10.3390/ijms25168730

**Published:** 2024-08-10

**Authors:** Marko Zlendić, Ema Vrbanović Đuričić, Koraljka Gall Trošelj, Marko Tomljanović, Kristina Vuković Đerfi, Iva Z. Alajbeg

**Affiliations:** 1 Department of Removable Prosthodontics, University of Zagreb School of Dental Medicine, Gundulićeva 5, 10000 Zagreb, Croatia; mzlendic@sfzg.unizg.hr; 2 Laboratory for Epigenomics, Division of Molecular Medicine, Ruđer Bošković Institute, 10000 Zagreb, Croatia; troselj@irb.hr (K.G.T.); marko.tomljanovic@irb.hr (M.T.); 3 Laboratory for Personalized Medicine, Division of Molecular Medicine, Ruđer Bošković Institute, 10000 Zagreb, Croatia; kvukovic@irb.hr; 4 Department of Dentistry, Clinical Hospital Center Zagreb, 10000 Zagreb, Croatia

**Keywords:** inflammation, cytokines, interleukin-8, single nucleotide polymorphism, chronic pain, temporomandibular disorders

## Abstract

This case-control study investigated single nucleotide polymorphism (SNP) genotypes (CXC motif chemokine ligand 8 (*CXCL8*): rs2227306 and rs2227307 and tumor necrosis factor (*TNF*): rs1800629) in 85 patients with pain-related temporomandibular disorders (TMDp) and 85 controls to explore their associations with TMDp presence, pain intensity (low/high), and the presence of chronic arthralgia/myalgia. TMDp was diagnosed using a validated protocol, and polymorphisms were genotyped from buccal mucosa swabs using TaqMan assays. High pain intensity individuals had an increased risk for carrying minor allele “G” (rs2227307) and “T” (rs2227306) compared to controls (76% vs. 55.3%, *p* = 0.012; 72% vs. 54.1%, *p* = 0.030, respectively). Carriers of the minor allele “G” (rs2227307) were more prevalent in TMDp patients with arthralgia compared to controls (70.30% vs. 55.30%, *p* = 0.037). According to logistic regression, the most important predictors for high pain intensity were minor allele “G” of rs2227307 (OR 2.435, 95% CI 1.123–5.282), increasing age (OR 1.038, 95% CI 1.002–1.075), and female sex (OR 4.592, 95% CI 1.289–16.361). The explored gene polymorphisms were not significant risk factors for TMDp presence. These findings highlight the importance of genetic variations, particularly rs2227307, in understanding the diverse clinical manifestations of temporomandibular disorders.

## 1. Introduction

Cytokines are small intracellular proteins primarily secreted by immune cells. These molecules bind to cytokine receptors on cell surfaces, initiating a cellular response and playing crucial roles in various physiological processes. One of the earliest discovered and extensively researched cytokines is interleukin-8 (IL-8), a chemotactic factor in inflammatory response [1]. It is encoded by the CXC motif chemokine ligand 8 gene (*CXCL8*), which consists of four exons and three introns located on chromosome 4q, with predominant expression in the bone marrow. Although certain genetic variations in *CXCL8* have been linked to more severe pain in lung cancer patients, similar associations between *CXCL8* variations and chronic pain disorders have not yet been investigated [2]. However, it has been observed that the salivary concentration of IL-8 is elevated in patients with temporomandibular disorders (TMD), a chronic and painful condition affecting the masticatory muscles and temporomandibular joints (TMJ) [3,4,5]. Comparable findings have been reported in patients with fibromyalgia, a condition that shares a pathophysiological background with TMD. Fibromyalgia patients had elevated serum levels of IL-8 compared to healthy controls [6].

Cytokines are commonly associated with inflammation. While the role of inflammation in the pathophysiology of TMD is highly debated, it is thought to play a significant role in a specific subgroup of TMD patients who have arthralgia. The hypothesis suggests that the infiltration of inflammatory cells, facilitated by synovial fluid, may contribute to the pathological destruction of TMJ and cartilage [7]. Synovial fluid analysis of TMD patients revealed the presence of IL-8 and tumor necrosis factor-alpha (TNF-α), markers absent in healthy controls [8]. Also, elevated levels of TNF-α were detected in the TMJ synovial fluid of patients with pain-related TMD (TMDp) compared to those with only internal derangements of TMJ [9]. On the other hand, other studies did not find differences in synovial fluid cytokine levels between TMD patients and healthy controls [10].

Although altered levels of proinflammatory cytokines have been identified in the synovial fluid of TMD patients, the underlying mechanisms are still poorly understood [11]. This is possibly due to a lack of clear evidence linking the severity of symptoms, such as pain, to the pathological destruction observed in the magnetic resonance imaging of TMJ [12]. Rather than solely attributing TMD symptoms to the inflammatory destruction of TMJ, it is more likely that another mechanism contributes to the observed association between TMD and elevated cytokine levels. Various intermediate phenotypes characterize TMD, primarily altered pain perception and the psychological response to stress [13,14]. Therefore, a possible mechanism involving cytokines likely includes their influence on altered nociceptive function, particularly considering the variations in pain sensitivity among patients. So, cytokine levels in synovial fluid are potential diagnostic indicators that could provide insights into TMD development [15].

Studies have shown that pain perception is affected by persistently high levels of proinflammatory cytokines, suggesting that genetic variations in cytokine-encoding genes might contribute to different phenotypes of TMD [16]. Variations in genes, specifically single nucleotide polymorphisms (SNPs), within proinflammatory genes, could influence pain sensitivity [17]. Another possibility is mediation through gene expression. A group of authors examined the gene expression of proinflammatory cytokines in the synovial fluid of TMD patients. They found higher mRNA levels for IL-1β, which belongs to the same subfamily of cytokines as IL-8 and TNF-α [18]. Gene polymorphisms might alter individual gene expression, which could subsequently influence the synthesis and biological activity of inflammatory cytokines. Since multiple SNPs likely have a combined influence on the clinical manifestations of TMDp, identifying those involved is crucial. In our previous study, we found that specific SNPs in the *COMT* and *OPRM1* genes were linked to poorer treatment responses in TMDp patients [19]. Identifying these genetic markers could improve our ability to predict treatment outcomes. Additionally, pinpointing potential indicators (such as SNPs) of pain susceptibility could have important clinical implications, making it easier to identify patients who are likely to have more severe symptoms.

Despite being primarily associated with disorders affecting TMJ, research has suggested a possible connection between cytokines and TMD myalgia. It has been proposed that teeth clenching and/or grinding affects blood flow, releasing proinflammatory compounds into the muscle [20]. Intense chewing has been linked to fatigue, soreness, and lower pressure pain thresholds in the masticatory muscles [21]. Patients suffering from myalgia had elevated levels of intramuscular IL-8. Furthermore, IL-8 levels rose in response to teeth clenching [22]. Although no direct link between TMD pain and inflammation has been discovered, it is conceivable to hypothesize that proinflammatory cytokines alter peripheral sensitization pathways and are thus involved in the pathophysiology of pain-related TMD.

In an attempt to further clarify whether gene variations, particularly SNPs within proinflammatory cytokine genes, may influence TMD presence and severity, the primary aim of this study was to compare the distribution of genotypes for specific SNPs in genes encoding proinflammatory cytokines (*CXCL8*: rs2227306 and rs2227307, and tumor necrosis factor (*TNF*): rs1800629) between patients with pain-related TMD and healthy control subjects. Additionally, this study sought to evaluate the potential predictive value of these SNPs for TMD-related pain and its intensity. Another objective was to explore possible associations between the selected SNPs and the presence of chronic arthralgia/myalgia in TMDp patients.

We hypothesized that certain genotypes of selected SNPs are associated with pain-related TMD presence and influence the severity of pain, differentiating between low and high intensities.

## 2. Results

### 2.1. Characteristics of Participants

The characteristics of the study participants are summarized in Table 1. The TMDp group included 85 subjects (76 women [89.4%] and 9 men [10.6%]) with an average age of 29.9 ± 11.1 years. This group can be described as follows:

- 44 participants (51.8%) had multiple diagnoses (41 women [93.2%] and 3 men [6.8%])
- arthralgia as the only diagnosis was present in 30 participants (35.3%) (28 women [93.3%] and 2 men [6.7%]).
- myalgia as the only diagnosis was present in 11 participants (12.9%) (7 women [63.6%] and 4 men [36.4%]).

Overall, myalgia (local myalgia, myofascial pain, or myofascial pain with referral) was observed in 64.7% of all TMDp subjects, while arthralgia was observed in 87.1% of all TMDp subjects.

The control group comprised 85 individuals (62 women [72.9%] and 23 men [27.1%]) who did not have muscle and/or joint symptoms and other TMD diagnoses, with an average age of 26.3 ± 7.7 years.

Women were significantly more represented in the TMDp group compared to the control group, but there was no significant age difference between the groups.

The average duration since pain onset in the TMDp group was 20.5 months, with over 58% reporting TMD-related pain in multiple areas. The Oral Behaviors Checklist showed a high frequency of oral parafunctions in both groups: 56.5% in the TMDp group and 58.8% in the control group, with no significant differences.

According to the GCPS score, we obtained the following results:

- 50 TMDp patients (58.8%) had high pain intensity (HPI), including 47 women.
- 35 TMDp patients (41.2%) had low pain intensity (LPI), including 29 women.

There were no significant differences in sex distribution between the HPI and LPI groups. However, individuals with a high frequency of oral parafunctions were more prevalent in the HPI group. Additionally, the HPI group had a significantly higher representation of people diagnosed with arthralgia compared to the LPI group.

### 2.2. Distribution of Genotype

Table 2 presents the genotype distribution of three SNPs in relation to the presence of pain (TMDp/CTR) and pain intensity (HPI/LPI).

There were no differences in the distribution of the rs1800629, rs2227306, and rs2227307 genotypes between HPI and LPI patients.

The distribution of the rs1800629 and rs2227306 genotypes was similar in TMDp patients and CTR subjects. However, the frequency of the rs2227307 (IL-8) genotype differed significantly between TMDp patients and CTR subjects. Patients carrying the minor allele G of rs2227307 were overrepresented in the TMDp group compared to healthy controls (66.4% vs. 55.3%, *p* = 0.041). This difference was even more pronounced when comparing the HPI TMDp group to control subjects. The HPI individuals had an increased risk of carrying the minor allele G of rs2227307 than control subjects (76% vs. 55.3%, *p* = 0.012, OR: 2.560, 95% CI: 1.177–5.570). Also, the HPI individuals showed an increased risk of carrying the minor allele T of rs2227306 compared to healthy controls (72% vs. 54.1%, *p* = 0.030, OR: 2.180, 95% CI:1.029–4.617).

### 2.3. Association between Selected Polymorphisms and the Presence of Chronic Arthralgia/Myalgia

There was no significant difference in the distribution of the rs2227306, rs2227307, and rs1800629 genotypes between TMDp patients with and without myalgia (*p* = 0.570, *p* = 0.373, *p* = 0.539, respectively) or between patients with and without arthralgia (*p* = 0.522, *p* = 0.449, *p* = 0174, respectively).

When evaluating the associations of SNPs within proinflammatory cytokine genes and particular disease entities related to TMDs (arthralgia and myalgia), we found no statistical link between the presence of specific SNPs in the proinflammatory cytokine genes and the occurrence of arthralgia and myalgia in TMDp patients (Table 3). Although no strong or statistically significant association was found overall, there was a very weak association (r = 0.143) between a specific genotype in the *CXCL8* gene and arthralgia.

**Figure 1:**
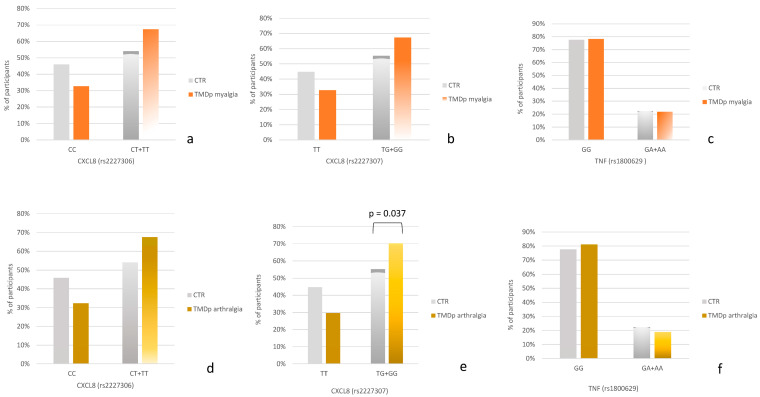
(**a**–**c**) Genotype distribution in control subjects (CTR) and temporomandibular disorders patients with myalgia (TMDp myalgia). (**d**–**f**) Genotype distribution in control subjects (CTR) and temporomandibular disorders patients with arthralgia (TMDp arthralgia).

When comparing TMDp patients with myalgia to control subjects (Figure 1a–c), the carriers of the minor allele G of rs2227307 as well as the carriers of the minor allele T of rs2227306 were slightly more prevalent in the TMDp myalgia group compared to healthy controls.

When comparing TMDp individuals with arthralgia to asymptomatic controls (Figure 1d–f), a significant association was observed for the *CXCL8* gene. Specifically, the carriers of the minor allele G of rs2227307 were more prevalent in the group of TMDp patients with arthralgia compared to healthy controls (70.3% vs. 55.3%, *p* = 0.037) (Figure 1e).

No association was observed between the genotype distribution of rs1800629 and the presence of chronic arthralgia or myalgia.

### 2.4. Risk Factors Associated with TMDp Presence

A logistic regression model was employed to determine the association of specific genetic polymorphisms with pain-related TMD, considering the age, sex, and frequency of oral behaviors as potential confounders or effect modifiers. Due to the strong association between rs2227307 and rs2227306 (r = 0.913, *p* = 0.001), logistic regression results are presented for rs2227307 (Model I) and rs2227306 (Model II). The probability of TMDp was significantly higher for the female sex (*p* = 0.007) and older age (*p* < 0.005), whereas SNP genotypes and the frequency of oral behaviors were not significant variables in this model of analysis (Table 4). The explanatory quality of this model, as indicated by the Nagelkerke R^2^, was 0.132.

### 2.5. Risk Factors Associated with Pain Intensity

Another logistic regression model was employed to determine the association of specific genetic polymorphisms with pain-related TMDs, considering the age, sex, and frequency of oral behaviors as potential confounders or effect modifiers. Due to the strong association between rs2227307 and rs2227306 (r = 0.913, *p* = 0.001), logistic regression results are presented separately for rs2227307 (Model I) and rs2227306 (Model II) as predictors of high pain intensity (Table 5).

According to logistic regression analysis, the probability of HPI was significantly higher in patients carrying the minor allele G of rs2227307 (OR 2.435, 95% CI 1.123–5.282, *p* = 0.024), older patients (OR 1.038, 95% CI 1.002–1.075, *p* = 0.036), and female patients (OR 4.592, 95% CI 1.289–16.361, *p* = 0.019). The explanatory quality (Nagelkerke R^2^) of this model was 0.158.

When rs2227306 was introduced in the analysis, the results showed that increasing age (OR 1.039, 95% CI 1.003–1.075, *p* = 0.032) and female sex (OR 4.568, 95% CI 1.284–16.250, *p* = 0.019) were positively associated with HPI, whereas *IL-8* polymorphism rs2227306 was not significant. The explanatory quality (Nagelkerke R^2^) of this model was 0.145.

## 3. Discussion

While the impact of cytokines on TMDs has been previously discussed, our results are the first to highlight the role of genetic polymorphisms in genes encoding proinflammatory cytokines. Although none of the examined SNPs predicted the presence of TMDp, patients carrying the G allele of SNP rs2227307 had more severe pain intensity. The model’s R^2^ value of 0.158 suggests a weak influence, which should be considered when interpreting these results. Additionally, although SNPs did not predict TMDp, age and sex had a weak influence on its presence (R^2^ = 0.132).

Other noteworthy results revealed a significantly higher prevalence of individuals carrying the minor allele of rs2227307 in the TMDp group compared to the control group. This difference was even more pronounced when TMD patients were stratified by pain intensity. The odds ratio of 2.560 indicates that TMDp individuals with high pain intensity were approximately 2.56 times more likely to carry the minor allele G of rs2227307 than control subjects. Also, the odds ratio of 2.180 indicates that TMDp individuals with high pain intensity were approximately 2.18 times more likely to carry the minor allele T of rs2227306 than control subjects. The TMDp group with high pain intensity showed a significantly higher prevalence of the minor alleles G (of rs2227307) and T (of rs2227306) compared to control subjects. This indicates an increased genetic susceptibility in individuals with high pain intensity associated with TMD for these specific SNPs. The odds ratios and confidence intervals support the robustness of these findings, pointing to a potential genetic marker for TMD-related pain intensity.

Although no strong or statistically significant association was found between SNPs in proinflammatory cytokine genes and specific TMD-related conditions such as arthralgia and myalgia, there was a very weak association between a specific genotype in the *CXCL8* gene and arthralgia. This weak association indicates that while the correlation is not strong enough to be conclusive or significant, it might suggest a potential area for further research. The near-significant *p*-value of 0.06 and the weak correlation (r = 0.143) suggests that with a larger sample size or more refined study design, this association might become clearer. Researchers can explore this SNP further, potentially in combination with other genetic or environmental factors, to better understand its role in arthralgia.

When the association of specific TMDp diagnoses and polymorphisms was analyzed, carriers of the minor allele rs2227307 were significantly more prevalent in patients with arthralgia compared to the control group. Carriers of the minor allele rs2227306 were also more prevalent in patients with arthralgia compared to the control group, although this trend was not statistically significant (Figure 1d–f). A similar trend was observed when comparing participants with myalgia to the control group. Minor allele carriers of rs2227306 and rs2227307 were more prevalent in patients with myalgia, but the differences were not statistically significant (Figure 1a–c). It is plausible that these differences could become significant with a larger sample size. Additionally, it indicates that the risk factor for TMDp, considering these SNPs, was being a carrier of the minor allele. Interestingly, the distribution of the rs1800629 (*TNF*) polymorphism was similar between both arthralgia patients and control subjects, as well as between myalgia patients and control subjects, indicating that this polymorphism might not be associated with the pathophysiology of TMDp.

Since significant differences were observed only between the arthralgia and control groups for rs2227307 (*CXCL8*), our research underscores the role of inflammatory cytokines in arthralgia. SNPs of the *CXCL8* have not yet been studied in the context of orofacial chronic pain. However, research has demonstrated that SNP rs2227307 is associated with more severe periodontal disease, a condition linked to specific types of oral pain [23]. Both orofacial chronic pain and periodontal disease are chronic and multifactorial conditions. The *CXCL8* SNP, particularly, rs2227307, appears to influence the clinical manifestations of periodontitis. Our study suggests that this SNP may contribute to the severity of TMD symptoms as well, indicating a potential shared genetic influence on both conditions. Regarding the influence of cytokines on pain intensity, it has been demonstrated that TMD patients with higher pain intensity and longer duration of symptoms have increased blood levels of IL-8 compared to patients with lower pain intensity and shorter duration of symptoms [24]. This finding partially aligns with our results, which indicate that carrying the G allele of SNP rs2227307 is a predictor of more severe pain intensity. Also, certain neuroendocrine peptides, such as neuropeptide Y and serotonin, along with the proinflammatory cytokine interleukin-1 beta (IL-1β), were found at higher levels in the synovial fluids of TMD patients [25]. It has already been established that SNPs in gene encoding serotonin receptors (*HTR2A*) are involved in the genesis of TMD symptoms such as hyperalgesia [26]. This suggests a possible interaction between the nervous and immune systems in pain modulation, which may result in increased pain sensitivity.

Even though the exact effect of proinflammatory cytokines in chronic pain disorders is unknown, the evidence suggests that genetics play a role. This may occur through gene expression that influences biological pathways regulating pain perception and psychological status [16]. The results of the study by Ogura et al. show that synovial fibroblasts, after stimulation by IL-1β, expressed high levels of IL-8 due to the upregulation of *CXCL8*, resulting in a 35-fold increase in mRNA synthesis [18]. The complexity of this mechanism becomes even more pronounced when genetic variations, such as SNPs, are considered. Our research found that carriers of the G allele of SNP rs2227307 in *CXCL8* were associated with TMDp and predicted higher pain intensity. This highlights the need for further investigation into this relationship. For instance, examining IL-8 levels in saliva, serum, or synovial samples and their association with *CXCL8* expression could provide valuable insights into the mechanisms of chronic pain disorders and the role of proinflammatory cytokines [6,18].

Our study has several limitations, one of which is the potential bias from not matching the sexes between the CTR and TMDp groups. To address this, our statistical analysis accounted for sex and other variables as possible confounders or effect modifiers. Additionally, our control group represents the general population, while TMD-prone patients were mostly women. Although our sample size was appropriate based on power analysis, larger studies would provide more reliable insights and definitive results. Regardless of these limitations, it is important to note the novelty of this study’s results.

## 4. Materials and Methods

### 4.1. Study Design and Participants

This case-control study was conducted at the University of Zagreb’s School of Dental Medicine, adhering to the ethical standards outlined in the Declaration of Helsinki. The research protocol received approval from the Ethics Committee of the University of Zagreb’s School of Dental Medicine (05-PA-30-VIII-6/2019). The trial was registered on 5 January 2021 at ClinicalTrials.gov (NCT046). The study protocol was developed following the guidelines of Strengthening the Reporting of Genetic Association Studies (STREGA): An Extension of the STROBE Statement, which controls the reporting of observational genetic studies [27].

Based on the calculation for the sample size for a case-control study with an equal ratio of participants (1:1) and considering a disorder prevalence of approximately 5%, as evidenced in prior research, a statistical error of 5% (α = 0.05), and a power of 80% for the dominant inheritance model, a total of 150 participants were deemed necessary with 75 allocated to each of the two groups [28,29].

Therefore, 85 patients were included in the patient group. They were recruited from individuals referred to the Department of Dentistry at the Clinical Hospital Center Zagreb between January 2020 and September 2022.

**Inclusion criteria.** Eligible participants were required to be over 18 years old and diagnosed according to the Diagnostic Criteria for Temporomandibular Disorders (DC/TMD) with TMDp, exhibiting myalgia (characterized by pain and dysfunction originating from masticatory muscles) and/or arthralgia (manifesting as spontaneous pain felt in the temporomandibular joint (TMJ) region, along with pain upon palpation of the TMJ’s lateral pole on the same side), with an average pain rating exceeding 30 mm on a visual analog scale (VAS), and symptoms persisting for more than three months [30].

**Exclusion criteria.** The general exclusion criteria comprised individuals under the age of 18, symptoms associated with pathologies in other areas of the orofacial region, acute pain (present for less than three months before the initial examination), history of TMJ trauma, headaches not related to TMDs (according to the International Classification of Headache Disorders, i.e., ICDH II), pain attributed to systemic illnesses, cancer, fibromyalgia, and confirmed psychiatric conditions. Patients consistently taking medications that might alter their consciousness and experience of pain, such as opioids, anticonvulsants, and antidepressants, as well as patients diagnosed solely with pain-free joint clicking and crepitation were also excluded.

Furthermore, the control group (CTR) consisted of 85 healthy individuals who did not have temporomandibular disorders (TMDs) and did not report any past or ongoing orofacial discomfort in their medical history. The control group comprised employees and patients of the Department of Dentistry at the Clinical Hospital Center Zagreb and students and faculty members from the University of Zagreb’s School of Dental Medicine. All participants belonged to the Caucasian ethnic group originating from Central and Southeast Europe. Before the commencement of this study, all participants were provided with detailed information regarding the study procedures and provided written informed consent.

### 4.2. Diagnosis of Temporomandibular Disorders

The evaluation and clinical examination of participants as well as the final diagnosis of painful TMDs were performed by skilled and experienced examiners (I.Z.A., E.V., and M.Z.) utilizing the validated Croatian version of the DC/TMD. The DC/TMD is a standardized and comprehensive tool used to diagnose and classify TMDs. It provides a dual-axis approach to ensure a thorough assessment of both the physical (Axis I) and psychosocial (Axis II) aspects of TMDs [30].

To ensure accuracy and consistency among examiners, three clinicians (I.Z.A., E.V., and M.Z.) conducted repeated clinical examinations on ten randomly selected subjects to evaluate TMD signs and symptoms based on the DC/TMD criteria. The analysis revealed no significant differences in the results (*p* > 0.05). Additionally, the agreement level between the examiners’ assessments was satisfactory, with kappa values ranging from 0.82 to 0.85.

The clinical assessment included palpation of the masticatory muscles and TMJs, examination of jaw movements, and analysis of TMJ sounds. To confirm a diagnosis of a pain-related temporomandibular disorder (TMDp) (either myalgia, arthralgia, or both), patients needed to verify experiencing pain in TMJs and/or masticatory muscles.

**Diagnosis of myalgia.** To diagnose myalgia, patients had to experience regional pain influenced by jaw movement, function, or parafunction. The examiner had to verify that the pain originated from the masticatory muscles, and patients had to confirm both the location and familiarity of the pain felt during jaw opening and palpation. The subtypes of myalgia were not considered for this research [30].

**Diagnosis of arthralgia.** To diagnose arthralgia, patients had to experience regional pain influenced by jaw movement, function, or parafunction. The examiner had to confirm that the pain was in TMJs, and patients had to verify the location and familiarity of the pain felt during jaw opening and palpation [30].

Additionally, as a part of the TMD assessment, participants completed a series of questionnaires as part of the DC/TMD standardized protocol. Pain intensity and pain-related disability were assessed with the Graded Chronic Pain Scale, and oral behavioral habits (parafunction) were evaluated with the Oral Behaviors Checklist.

#### 4.2.1. Pain Assessment

To be included in this study, participants needed to have experienced chronic pain for more than three months and have an average pain rating exceeding 30 mm on the visual analog scale (VAS) at the time of the first examination. Additionally, they must not have used any medication that alters pain perception for an extended period prior to the initial examination, as specified in the inclusion criteria. By using the VAS to determine spontaneous pain, we identified a cohort of individuals with TMDs who were experiencing moderate to severe pain. This selection criterion limits the generalizability of our findings to the broader TMD population, which includes individuals with varying degrees of pain severity, including those with mild or slight discomfort [31,32].

Once participants were deemed eligible for this study, their pain was further evaluated using a validated DC/TMD protocol. A questionnaire that assesses chronic pain within this protocol is the Graded Chronic Pain Scale (GCPS). It is routinely used to provide detailed information about a patient’s chronic pain. Specifically, we analyzed variables such as current pain, worst pain, average pain, and characteristic pain intensities in this study. Additional information regarding the time since pain onset and whether patients experienced pain in multiple areas was also obtained from the DC/TMD protocol.

**Graded Chronic Pain Scale (GCPS).** The Graded Chronic Pain Scale is a component of the DC/TMD, specifically within Axis II, which focuses on TMDs’ psychosocial and behavioral aspects. It assesses current, worst, and average pain levels. Each item is rated on a scale of 0 to 10, with 0 indicating no pain and 10 indicating the worst pain imaginable. Characteristic Pain Intensity (CPI) is a key measure within the GCPS. It provides a quantitative assessment of the overall pain experience of a patient. The CPI represents the mean pain intensity reported for current, worst, and average pain levels over the past 30 days [30].

#### 4.2.2. Oral Behavior Assessment

**Oral Behavior Checklist (OBC).** The Oral Behavior Checklist is a diagnostic tool to evaluate patients’ parafunctional oral habits and behaviors. It is a part of Axis II in the DC/TMD. These habits can include clenching, grinding (bruxism), nail biting, lip and cheek biting, and other repetitive actions that may affect dental and oral health. The OBC is typically administered as a self-report questionnaire, where patients or their caregivers answer a series of questions about specific oral behaviors. Each question assesses the frequency of a particular behavior using a Likert scale (0 = Never, 1 = Rarely, 2 = Sometimes, 3 = Often, and 4 = Very Often). The checklist consists of 21 items: the first two items evaluate activities during sleep, while the remaining items focus on behaviors occurring during waking hours [30,33].

### 4.3. Participant Categorization and Cut-Off Values

This study initially divided participants into two main groups: those diagnosed with TMDp and a CTR group that did not experience pain. Within the TMDp group, further categorization was based on pain intensity, assessed using the GCPS questionnaire. Patients with a CPI score below 50 were grouped into the low pain intensity (LPI) category, while those scoring 50 or above were placed in the high pain intensity (HPI) category [34].

To examine parafunctional behaviors, participants were categorized following the guidelines outlined by Ohrbach and Knibbe in the DC/TMD Scoring Manual for Self-Report Instruments (ACTA). Those exhibiting high-frequency oral behavior had OBC sum scores ranging from 25 to 84, while individuals demonstrating low-frequency oral behavior had OBC sum scores ranging from 1 to 24 [35].

### 4.4. Selection of SNPs and Gene IDs

The CXC motif chemokine ligand 8 gene (*CXCL8*; Gene ID: 3576) encodes the protein from the CXC chemokine family commonly referred to as interleukin 8. The gene is located on 4q13.3 and consists of four exons and three introns. In this research, we examined two SNPs, namely, rs2227306 and rs2227307 in *CXCL8.*

Polymorphism rs2227306 (NC_000004.12:73741337:C:T) is located in the non-coding region of *CXCL8* and can occur as cytosine (C) or thymine (T).

Polymorphism rs2227307 (NC_000004.12:73740951:T:G) is located in the non-coding region of *CXCL8* and can occur as thymine (T) or guanine (G).

The tumor necrosis factor gene (*TNF*; Gene ID: 7124) encodes the protein from the TNF superfamily called TNF-alpha. The gene is located on 6p21.33 and consists of four exons and three introns. In this research, we examined SNP rs1800629 in *TNF*.

Polymorphism rs1800629 (NM_000594.3(*TNF*):c.-488G>A) is located in the promoter region of *TNF*, and it can occur as guanine (G) or adenine (A).

Individuals homozygous for adenine, guanine, cytosine, or thymine alleles were marked as having AA, GG, CC, or TT genotypes, respectively. Heterozygous individuals were marked as having CT, TG, or GA genotypes.

### 4.5. Sample Collection, DNA Extraction, and Genotyping

Following a clinical examination, each participant underwent buccal swab collection using a soft nylon bristle brush (Cytology Rambrush, Mirandola, Italy). Buccal swab samples were stored in phosphate-buffered saline (PBS)-filled microtubes (Eppendorf, Hamburg, Germany) and preserved at −20 °C for subsequent DNA extraction. According to the manufacturer’s protocol, genomic DNA extraction was performed using the commercially available QIAamp^®^ DNA Mini Kit (QIAGEN™, Venlo, The Netherlands). The quality of the extracted DNA, which was satisfactory for all samples, was assessed using 1% agarose gel electrophoresis with ethidium bromide staining. The final DNA concentration was determined by measuring the absorbance at 260 and 280 nm using a NanoDrop™ spectrophotometer (Thermo Fisher Scientific, Waltham, MA, USA). The yield of the extracted DNA ranged from 10 to 30 μg/μL, which was adequate for SNP genotyping.

Three SNPs (rs2227306—C_11748169_10, rs2227307—C_11748168_10, and rs1800629—C_7514879_10) in two candidate genes (*CXCL8* and *TNF*) encoding inflammation-related cytokines were genotyped. In 0.2 mL optical tubes (Applied Biosystems, Waltham, MA, USA), we prepared a reaction mixture with the following components: 1 μL DNA, 1 μL Taqman™ Genotyping Assays (Applied Biosystems, Waltham, MA, USA), 10 μL TaqPath™ ProAmp™ Master Mix (Applied Biosystems, Waltham, MA, USA), and 8 μL Milli-Q water. The total volume per sample was 20 μL. The reaction was carried out using the 7300 Real-Time PCR System (Applied Biosystems, Waltham, MA, USA) under the following conditions: 2 min at 50 °C, 10 min at 95 °C, followed by 40 cycles of 15 s at 95 °C, and a final step of 1 min at 60 °C. In addition to the negative “no template control” and positive controls, genotyping quality control included the use of blind technical duplicates to detect discrepancies, removal of samples with low DNA quality, and evaluation of deviations from the Hardy–Weinberg equilibrium.

### 4.6. Statistical Analysis

Data processing and statistical analysis were performed using IBM SPSS Statistics for Windows, Version 26.0 (Armonk, NY, USA: IBM Corp). To verify a normal distribution of the numerical variables, the Shapiro–Wilk test was used. The Mann–Whitney U test and the chi-square test were used to evaluate the differences in demographic and clinical characteristics between groups.

Differences in the frequencies of genotypes between the groups were analyzed using the chi-square test after fitting for the Hardy–Weinberg equilibrium. The risks associated with carrying certain genotypes were calculated as the odds ratio (OR) with a 95% confidence interval (CI).

The assessment was conducted using a dominant genetic model, considering the effect of carrying one or both minor alleles. In this model, the major allele served as the reference allele, while the minor allele was identified as the risk allele.

Spearman correlations were used to test associations of single nucleotide polymorphisms within proinflammatory cytokine genes and particular disease entities related to the TMD (arthralgia and myalgia).

Finally, logistic regression analysis was used to reveal the factors associated with pain-related TMDs.

Values of *p* < 0.05 were considered statistically significant, and the risks associated with individual alleles and genotypes were calculated as the odds ratio (OR) with a 95% confidence interval (CI).

## 5. Conclusions

Individuals carrying the minor allele of rs2227307 (*CXCL8*) were more prevalent in the pain-related TMD group than healthy controls. They were also represented more often in the group of patients experiencing higher levels of pain compared to controls. Furthermore, carriers of the same minor allele were significantly more prevalent among patients with arthralgia compared to the control group. Patients carrying the GG and TG genotypes of rs2227307, older patients, and female patients are strong risk factors for high pain intensity related to TMDs.

These findings highlight the increasing importance of genetic variations, specifically the inflammation-related polymorphism rs2227307, in understanding the diverse clinical manifestations of temporomandibular disorders. Identifying SNPs involved in chronic pain processes is essential for understanding the incidence, onset, and persistence of these complex disorders. This understanding offers the potential to deepen insights into pain-related temporomandibular disorders, especially symptoms related to high pain intensity and arthralgia. It could lead to the development of more precise and effective diagnostics for individuals affected by these conditions and potentially help to develop genetic tests that could customize the treatment for each patient.

## Figures and Tables

**Table 1:** Socio-demographic and clinical characteristics of patients with pain-related temporomandibular disorders and healthy controls.

Variable			LPI (n = 35)	HPI (n = 50)	LPI + HPI (n = 85)	CTR (n = 85)
Sex	Female	n (%)	29 (82.9%)	47 (94%)	76 (89.4%)	62 (72.9%)
	Male	n (%)	6 (17.1%)	3 (6%)	9 (10.6%)	23 (27.1%)
		*p* ^a^	0.152		**0.010**	
Age	Female	Mean (SD)	28.69 (10.01)	30.02 (11.66)	29.51 (11.01)	26.15 (7.71)
		*p*	0.748		0.067	
	Male	Mean (SD)	33.33 (13.23)	34.67 (12.58)	33.78 (12.22)	26.52 (7.91)
		*p* ^b^	0.897		0.064	
Oral behaviors	Low frequency	n (%)	20 (57.1%)	17 (34%)	37 (43.5%)	35 (41.2%)
	High frequency	n (%)	15 (42.9%)	33 (66%)	48 (56.5%)	50 (58.8%)
		*p* ^a^	**0.046**		0.877	
Pain variables	Time since pain onset (months)	mean (SD)	17.8 (18.7)	22.4 (24.8)	20.5 (22.5)	-
		*p* ^b^	0.438		NA	
	Pain in more than one area	n (%)	18 (51.4%)	32 (64%)	50 (58.8%)	-
		*p* ^a^	0.271		NA	
Pain diagnosis	Arthralgia	n (%)	27 (77%)	47 (94%)	74 (87.1%)	-
			**0.023**		NA	
	Myalgia	n (%)	22 (62.9%)	33 (66%)	55 (64.7%)	-
			0.471		NA	

^a^ Chi-square test; ^b^ Mann–Whitney U test; High pain intensity group, HPI; Low pain intensity group, LPI; Control group—absence of TMD diagnosis, CTR; Number of participants, n; Standard deviation, SD; and *p*-value, *p*. Significant *p*-values are marked in bold.

**Table 2:** Genotypic frequencies according to the presence of pain (TMDp/CTR) and pain intensity (LPI/HPI)—dominant model.

	Pain Presence				Pain Intensity			
	TMDp (n = 85)		CTR (n = 85)		LPI (n = 35)		HPI (n = 50)	
rs1800629 (*TNF*) Wild: G Minor: A	GG	GA + AA	GG	GA + AA	GG	GA + AA	GG	GA + AA
n (%)	67 (78.8%)	18 (21.2%)	66 (77.6%)	19 (22.4%)	26 (74.3%)	9 (25.7%)	41 (82%)	9 (18%)
*p*-value (OR, 95% CI)	0.500 (0.933, 0.450–1.934)				0.277 (0.634, 0.233–1.806)			
rs2227306 (*CXCL8*) Wild: C Minor: T	CC	CT + TT	CC	CT + TT	CC	CT + TT	CC	CT + TT
n (%)	28 (32.9%)	57 (67.1%)	39 (45.9%)	46 (54.1%) ^**A**^	14 (40%)	21 (60%)	14 (28%)	36 (72%) **^A^**
*p*-value (OR, 95% CI)	0.058 (1.726, 0.927–3.214)				0.178 (1.714, 0.686–4.283)			
rs2227307 (*CXCL8*) Wild: T Minor: G	TT	TG + GG	TT	TG + GG	TT	TG + GG	TT	TG + GG
n (%)	26 (30.6%)	59 (69.4%)	38 (44.7%)	47 (55.3%) ^**B**^	14 (40%)	21 (60%)	12 (24%)	38 (76%) **^B^**
*p*-value (OR, 95% CI)	**0.041 (1.835, 1.978–3.442)**				0.091 (2.111, 0.827–5.390)			

Patients with painful temporomandibular disorders—presence of myalgia, arthralgia, or both, TMDp; Control group—absence of TMD, CTR; High pain intensity group, HPI; Low pain intensity group, LPI; Number of participants, n; Tumor necrosis factor, *TNF*; CXC Motif Chemokine Ligand 8 gene, *CXCL8*; Odds ratio, OR; and Confidence interval, CI. Significant *p*-values are marked in bold. ^A^ Pearson chi-square result of the comparison of CT + TT genotypic frequencies of CTR vs. HPI *p*-value (OR, 95% CI): 0.030 (2.180, 1.029–4.617). ^B^ Pearson chi-square result of the comparison of TG + GG genotypic frequencies of CTR vs. HPI *p*-value (OR, 95% CI): 0.012 (2.560, 1.177–5.570).

**Table 3:** Correlations of SNPs in proinflammatory cytokine genes with TMDp-related arthralgia and myalgia.

		rs1800629 (*TNF*) (Homozygous GG_0; Heterozygous GA + AA_1)	rs2227306 (*CXCL8*) (Homozygous CC_0; Heterozygous CT + TT_1)	rs2227307 (*CXCL8*) (Homozygous TT_0; Heterozygous TG + GG_1)
TMDp arthralgia (0 = no, 1 = yes)	r	−0.061	0.125	0.143
	*p*	0.432	0.103	0.060
TMDp myalgia (0 = no, 1 = yes)	r	0.001	0.094	0.070
	*p*	0.990	0.219	0.362

Temporomandibular disorders patients with myalgia, TMDp myalgia; Temporomandibular disorders patients with arthralgia, TMDp arthralgia; Tumor necrosis factor, *TNF*; CXC motif chemokine ligand 8 gene, *CXCL8; p*-value, *p*; Correlation coefficient, r.

**Table 4:** Binary logistic regression results for predictors of pain-related TMD.

	B	S.E.	*p*	OR	95% CI
**Model I**					
rs2227307 (*CXCL8*) (homozygous TT_0; heterozygous TG + GG_1)	0.551	0.335	0.100	1.735	0.900–3.345
oral behaviors (low_0; high_1)	−0.118	0.337	0.726	0.889	0.459–1.720
sex (male_0; female_1)	1.223	0.452	0.007 *	3.399	1.400–8.248
age	0.043	0.018	0.016 *	1.044	1.008–1.081
**Model II**					
rs2227306 (*CXCL8*) (homozygous CC_0; heterozygous CT + TT_1)	0.491	0.332	0.139	1.634	0.853–3.131
oral behaviors (low_0; high_1)	−0.115	0.336	0.733	0.892	0.461–1.724
sex (male_0; female_1)	1.218	0.453	0.007 *	3.380	1.392–8.206
age	0.044	0.018	0.014 *	1.045	1.009–1.082

No standardized variable coefficient, B; Standard error, S.E.; *p*-value, *p*; Odds ratio, OR; Confidence interval, CI; and CXC motif chemokine ligand 8 gene, CXCL8. Significant *p*-values are indicated by an asterisk (*).

**Table 5:** Binary logistic regression results for predictors of high pain intensity.

	B	S.E.	*p*	OR	95% CI
**Model I**					
rs2227307 (*CXCL8*) (homozygous TT_0; heterozygous TG + GG_1)	0.890	0.395	0.024 *	2.435	1.123–5.282
oral behaviors (low_0; high_1)	0.541	0.380	0.154	1.718	0.816–3.620
sex (male_0; female_1)	1.524	0.648	0.019 *	4.592	1.289–16.361
age	0.037	0.018	0.036 *	1.038	1.002–1.075
**Model II**					
rs2227306 (*CXCL8*) (homozygous CC_0; heterozygous CT + TT_1)	0.710	0.381	0.062	2.033	0.964–4.290
oral behaviors (low_0; high_1)	0.527	0.377	0.163	1.693	0.808–3.548
sex (male_0; female_1)	1.519	0.647	0.019 *	4.568	1.284–16.250
age	0.038	0.018	0.032 *	1.039	1.003–1.075

No standardized variable coefficient, B; Standard error, S.E.; *p*-value, *p*; Statistically significant, *; Odds ratio, OR; Confidence interval, CI; and CXC motif chemokine ligand 8 gene, CXCL8. Significant *p*-values are indicated by an asterisk (*).

## Data Availability

Data are contained within the article. Any data that may be relevant to this research are available from the corresponding author upon reasonable request.
